# GALLBLADDER POLYPS: CORRELATION AND AGREEMENT BETWEEN ULTRASONOGRAPHIC AND HISTOPATHOLOGICAL FINDINGS IN A POPULATION WITH HIGH INCIDENCE OF GALLBLADDER CANCER

**DOI:** 10.1590/0102-672020230002e1732

**Published:** 2023-05-08

**Authors:** Martin Inzunza, Maria Jesus Irarrazaval, Paloma Pozo, Fernando Pimentel, Fernando Crovari, Luis Ibañez

**Affiliations:** 1 Pontificia Universidad Católica de Chile, , Faculty of Medicine, Department of Digestive Surgery – Santiago, Chile;; 2 Pontificia Universidad Católica de Chile, Faculty of Medicine – Santiago, Chile.

**Keywords:** Gallbladder diseases, Gallbladder neoplasms, Cholecystectomy, laparoscopic, Ultrasonography, Polyps, Doenças da vesícula biliar, Neoplasias da vesícula biliar, Colecistectomia laparoscópica, Ultrassonografia, Pólipos

## Abstract

**BACKGROUND:**

Gallbladder polyps are becoming a common finding in ultrasound. The management has to consider the potential risk of malignant lesions.

**AIMS:**

The aim of this study was to analyze the ultrasound findings in patients undergoing cholecystectomy due to gallbladder polyps and compare them for histopathological findings (HPs).

**METHODS:**

Patients with an ultrasonographic diagnosis of gallbladder polyp and who underwent cholecystectomy from 2007 to 2020 were included in the study.

**RESULTS:**

A total of 447 patients were included, of whom 58% were women. The mean age was 45±12 years. The mean size of polyps in US was 7.9±3.6 mm. Notably, 9% of polyps were greater than 10 mm, and single polyps were significantly larger than the multiple ones (p=0.003). Histopathological findings confirmed the presence of polyps in 88.4%, with a mean size of 4.8±3.4 mm. In all, 16 cases were neoplastic polyps (4.1%), 4 of them being malignancies, and all were single and larger than 10 mm. We found a significant correlation between ultrasound and histopathological findings polyp size determination (r=0.44; p<0.001). The Bland-Altman analysis obtained an overestimation of the US size of 3.26 mm. The receiver operating characteristic (ROC) curve analysis between both measures obtained an area under the receiver operating characteristic curve (AUC) of 0.77 (95%CI 0.74–0.81). Ultrasound polyps size larger than 10 mm had an odds ratio (OR) of 8.147 (95%CI 2.56–23.40) for the presence of adenoma and malignancy, with a likelihood ratio of 2.78.

**CONCLUSIONS:**

There is a positive correlation and appropriate diagnostic accuracy between ultrasound size of gallbladder polyps compared to histopathological records, with a trend to overestimate the size by about 3 mm. Neoplastic polyps are uncommon, and it correlates with size. Polyps larger than 10 mm were associated with adenoma and malignancy.

## RESUMO

**RACIONAL:**

Os pólipos da vesícula biliar estão se tornando um achado comum na ultrassonografia (US). O manejo deve levar em consideração o risco de lesões malignas.

**OBJETIVOS:**

Analisar os achados da ultrassonografia em pacientes submetidos à colecistectomia por pólipos vesicais e compará-los com os achados histopatológicos.

**MÉTODOS:**

Foram revisados os prontuários médicos dos pacientes com diagnóstico ultrassonográfico de pólipo vesicular e submetidos à colecistectomia no período de 2007 a 2020.

**RESULTADOS:**

Foram incluídos no estudo 447 pacientes. A média de idade foi 45±12anos, sendo 58% mulheres. O tamanho médio dos pólipos na US foide 7,9±3,6mm. Nove por cento foram maiores que 10 mm, e os pólipos únicos encontrados foram maiores do que os múltiplos (p=0,003). A HP confirmou a presença de pólipos em 88,4%, tamanho médio 4,8±3,4mm. Dezesseis eram pólipos neoplásicos (4,1%) e quatro deles malignos, únicos e maiores que 10 mm. Foi encontrado correlação significativa entre a determinação do tamanho do pólipo ao ultrassonografia e histopatológicos (r=0,44; p<0,001). A análise de Bland-Altman obteve uma superestimação do tamanho do pólipo ao US em 3,26 mm. A análise da curva da característica de operação do receptor entre as duas medidas obteve uma área sob a curva curva da característica de operação do receptor (AUC) de 0,77 (IC95% 0,74–0,81). Pólipos ao ultrassonografia maiores que 10 mm apresentaram razão de chance (OR) de 8,147 (IC95% 2,56–23,40) para presença de adenoma e malignidade, com razão de verossimilhança de 2,78.

**CONCLUSÕES:**

Há uma correlação positiva e acurácia diagnóstica apropriada entre o tamanho dos pólipos da vesícula biliar por ultrassonografia em comparação com os achados histopatológicos, com uma tendência de superestimar o tamanho em cerca de 3 mm. Pólipos maiores que 10 mm foram associados a adenoma e malignidade.

## INTRODUCTION

Gallbladder polyps are an outgrowth of the mucosal wall of the gallbladder. They can be benign or malignant. Benign lesions can be classified as neoplastic (i.e., adenoma, lipoma, and leiomyoma) or non-neoplastic (i.e., cholesterol polyps, adenomyomas, inflammatory polyps, and hyperplastic polyps, among others). Malignant lesions commonly involve adenocarcinomas and metastases, as ICPN and non-neoplastic polyps harboring dysplasia are much less frequent^10^.

Most gallbladder polyps are discovered incidentally on abdominal ultrasound (US). Detection rates have increased due to the widespread use of ultrasonography. Epidemiological studies indicate a US prevalence of 4–5.6%. Its incidence in cholecystectomy is up to 13.8% according to the international series; meanwhile, it is around 0.6–1% in Chile^4,34^.

Accurate diagnosis is relevant due to the possibility that malignant neoplasms may develop from polyps or that malignant lesions may adopt polypoid morphologies on US, delaying their diagnosis and treatment. The incidence of gallbladder cancer is highest in South American countries such as Bolivia (14 per 100,000) and Chile (9.3 per 100,000), and other countries such as Thailand (7.4 per 100,000), South Korea (6.8 per 100,000), and Nepal (6.7 per 100,000)^32^. Chile has the highest mortality rate associated with gallbladder cancer in the world (7.8 per 100,000). In fact, gallbladder cancer is the third leading cause of death related to cancer in women in this country, with an estimated survival of less than 10% at 5 years. Furthermore, the prevalence of finding this neoplasm in cholecystectomies is 3.4% in women and 1.32% in men^32^.

Gallbladder cancer is more prevalent in women than in men. Classical risk factors of this pathology are gallstone disease, polyps, chronic infections of the gallbladder, obesity, alcohol, tobacco, elevated blood sugar, medications, and exposure to certain carcinogens^11,20^.

The objective of this study is to correlate US and histopathological findings in cholecystectomized patients due to gallbladder polyps in the past 13 years.

## METHODS

### Study design

A retrospective cohort study was conducted at the Clinical Hospital of the Pontificia Universidad Católica de Chile, Santiago, Chile. This study was approved by the Institutional Ethics Committee.

### Patient selection

We included all patients aged more than 18 years, who underwent cholecystectomy due to gallbladder polyps diagnosed by US, from 2007 to 2020, in a single university center. Incomplete medical records and gallbladder polyps diagnosed with other image-study than US were considered exclusion criteria.

### Variables and outcomes

Demographic data, diagnosis, and type of surgery were included. Preoperative US findings were compared to histological findings (i.e., type, size, number of polyps, and presence/absence of associated cholelithiasis). As the primary outcome, correlation and agreement in terms of size between US and histological findings were analyzed. As a secondary outcome, the prevalence of adenoma and adenocarcinoma was also analyzed.

### Statistical analysis

Statistical analysis was carried out using SPSS version 25 (IBM Corp., Armonk, New York). Student's t-test was applied for comparison of means, Mann-Whitney test was used for comparison of means without normal distribution, and chi-square test was used for comparison of proportions, as appropriate. Pearson's correlation coefficient, Bland-Altman analysis, and receiver operating characteristic (ROC) curve analysis were applied to assess correlation, agreement, and diagnostic accuracy, respectively. All data were expressed as median and interquartile range, or mean and standard deviation, as appropriate. A p-value <0.05 was considered statistically significant.

## RESULTS

A total of 15,835 cholecystectomies were performed from 2007 to 2020, and 734 of them were preoperative US diagnosis of gallbladder polyp. A total of 447 patients were included according to the inclusion and exclusion criteria, of whom 58.4% (n=261) were women, and the mean age was 45±12 years. Of all patients who underwent laparoscopic surgery, 11.6% (n=52) were associated with other procedures; the most common were hernia repair (n=25, 5.6%) and bariatric surgery (n=19, 4.3%).

### Ultrasonographic findings

The preoperative diagnosis of gallbladder polyps was performed with US in all patients (Table 1). A total of 45.9% (205 patients) had one polyp, while 54.1% (242 patients) had two or more polyps in the US. The mean size of the polyps was 7.9±3.6 mm.

**Table 1:** Ultrasound findings.

		n (%)	Mean size (SD) (mm)
**Number of polyps**			
	One polyp	205 (45.9)	8.1 (4.2)
	Two or more polyps	242 (54.1)	7.9 (3.1)
**Associated cholelithiasis**		68 (15.2)	7.3 (5.0)
	Microlithiasis/biliary sludge	38 (8.5)	–

SD: standard deviation.

Associated cholelithiasis was diagnosed in 68 patients (15.2%), with a mean size of 5.2±5.1 mm. Microlithiasis/biliary sludge was reported in 38 patients (8.5%).

### Histopathological findings

The histopathological analysis confirmed the presence of gallbladder polyps in 88.4% of the patients (n=395). The mean size of polyps was 4.8±3.4 mm. Associated cholelithiasis was diagnosed in 91 (23%) patients. Of note, 193 (48.9%) patients had one polyp, and 202 (51.1%) patients had two or more polyps (Table 2). Surgical indications in patients with no polyps described in the histopathological analysis are described in Table 3.

**Table 2:** Histopathological findings.

		n (%)
**Number of polyps**		
	One polyp	193 (48.9)
	Two or more polyps	202 (51.1)
**Associated cholelithiasis**		91 (23)
**Classification of polyps**		
	**Non-neoplastic**	379 (95.9)
	Cholesteroloris	72 (19.5)
	Cholesterol polyp	281 (71.1)
	Adenomyomas	14 (3.5)
	Other	12 (3)
	**Neoplastic**	16 (4.1)
	Adenoma	12 (3)
	Lymphoma*	1 (0.3)
	Adenocarcinoma	3 (0.8)

* Adenoma infiltrated by lymphoma.

**Table 3:** Surgical indications in patients with no polyps described in histopathological analysis (n=52).

Surgical indication	n (%)
Polyp greater than 1 cm in US	38 (73.1)
Abdominal pain	7 (13.5)
Simultaneous bariatric surgery	4 (7.7)
Pancreatitis	2 (3.8)
Associated cholelithiasis	1 (1.9)

Considering the classification of lesions^26^, 379 (95.9%) patients had non-neoplastic polyps, being cholesteric polyps (n=72, 19.5%) the most frequent one. In contrast, 16 (4.1%) patients had neoplastic polyps: 12 patients with adenoma diagnosis, one patient with an adenoma infiltrated by lymphoma, and three patients with adenocarcinoma diagnosis (Table 4). Of these three patients, one presented a T2N0M0 gallbladder cancer, and the other two had ICPN finding with high-grade dysplasia and microinvasive carcinoma.

**Table 4:** Characteristics of patients with gallbladder adenocarcinoma.

Patient	Sex	Age	Surgical indication	Comorbidities	Number of polyps in US	US polyp size (mm)	HP polyp size (mm)	Associated cholelithiasis
1	Male	74	US finding	Obesity	1	34	16.7	Yes
2	Female	38	Abdominal pain	No	1	28	14	No
3	Male	38	US finding	No	2	10	10	No

US: ultrasound; HP: histopathological findings.

### Correlation, agreement, and diagnostic accuracy

**Figure 1:**
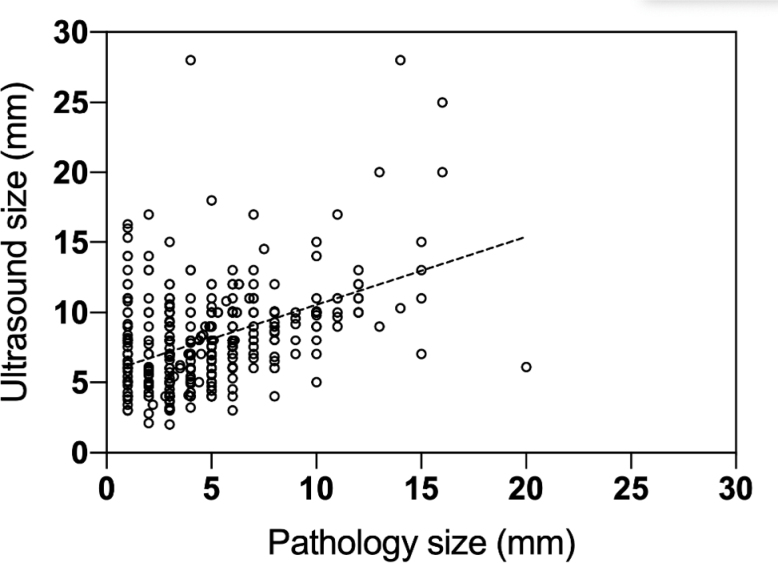
Correlation between ultrasound size (mm) and pathology size (mm).

Assessing the correlation between US size (mm) and pathology size (mm) measures of the polyps with the Pearson correlation coefficient, we obtained an r-value of 0.44 (p<0.001) (Figure 1).

**Figure 2:**
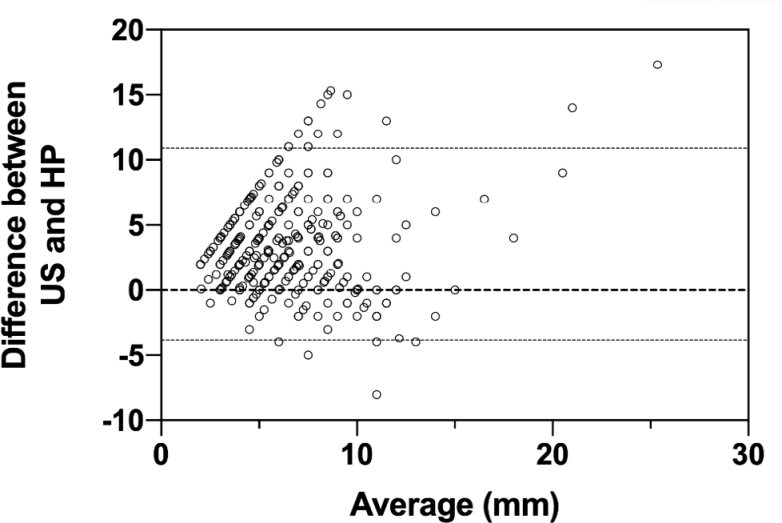
Bland-Altmann analysis between ultrasound size and histopathological measures.

US: ultrasound; HP: histopathological records; mm: millimeters.

Assessing the agreement between ultrasonographic and histopathological size measures of the polyps with the Bland-Altman analysis, we obtained a bias value of 3.26 mm. In consequence, ultrasonographic measures tend to overestimate the size of the gallbladder polyps of up to 3.26 mm. This trend is odd to find in polyps between 5 and 10 mm (Figure 2).

**Figure 3:**
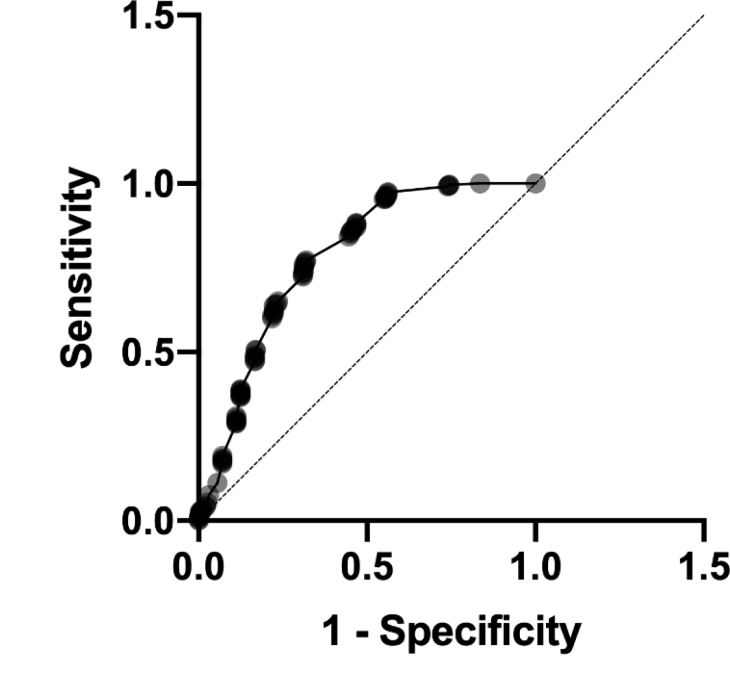
ROC curve analysis between ultrasound size and histopathological measures.

Furthermore, an ROC curve analysis was developed between sizes obtained with the US and histopathological measures. The area under the ROC curve (AUC) value was 0.77 (95%CI 0.74–0.81) (Figure 3).

ROC: receiver operator characteristic.

US polyps’ size larger than 10 mm also had an odds ratio (OR) of 8.147 (95%CI 2.56–23.40) for the presence of adenoma, with a likelihood ratio (LR) of 2.78. Meanwhile, a US polyp size measure between 5 and 10 mm had an OR of 0.254 (95%CI 0.08–0.79) for the presence of adenoma findings in the histopathological analysis, with an LR of 0.44 (Table 5).

**Table 5:** Odds ratio and likelihood ratio for adenoma presence.

US polyps’ size	OR for adenoma presence (95%CI)	LR for adenoma presence
5–10 mm	0.254 (0.08–0.79)	0.44
>10 mm	8.147 (2.56–23.40)	2.78

US: ultrasound; OR: odds ratio; CI: confidence interval; LR: likelihood ratio.

## DISCUSSION

Gallbladder polyps are an increasingly common finding on abdominal US scans^5,7^. In this case series, 88.4% of patients with preoperative US diagnosis of gallbladder polyps had confirmed polyps on histology, and the majority of them were multiple. In 23%, they were associated with lithiasis or microlithiasis. This finding constitutes an interesting piece of information because the association between cholesterolosis and gallbladder cancer is inverse, with 9.2 times less probability of having gallbladder cancer in patients with cholesterolosis^23^. Regarding the type of polyp, more than 90% were non-neoplastic polyps and 4.1% had neoplastic polyps. Among neoplastic polyps, 13 patients had an adenoma (one of them infiltrated by lymphoma), and three patients had an adenocarcinoma diagnosis. They constitute a percentage very similar to other series^16,23,34^.

In our study, the adenoma and adenocarcinoma prevalence (3.5 and 0.8%, respectively) are concordant with that reported in the literature. Other experiences describe adenoma rates of 4.9, 3.1, 7.2, and 8.85%^16,33^ and adenocarcinoma rates of 0, 0.05, 1.5, 1.8, and 1.9%^9,16,21,25^. This high variability may be explained by the different prevalence of gallbladder disease in the study populations^15^. The incidence of gallbladder cancer is higher in Asian and Latin-American countries and lower in the European population^2,4,14^. For example, in countries such as Chile and South Korea, the age-standardized gallbladder cancer rate is 9.3 and 6.8 per 100,000, respectively. In contrast, in African and European countries, it does not exceed 2.5 per 100,000^12^.

A Cochrane review published in 2018 described the sensitivity and specificity of US for detecting gallbladder polyps as 0.79 and 0.89, respectively^29^. Ostapenko et al. reported a 54% correlation between US and histopathological diagnosis for gallbladder polyps, and Ahmed et al. described a positive predictive value of 0.77 and a negative predictive value of 1.0 for the diagnosis of true and pseudo-gallbladder polyps using ultrasonography^1,21^. A most recently published systematic revision found that for true polyps, transabdominal ultrasonography had a sensitivity of 83.1%, a specificity of 96.3%, a positive predictive value of 14.9%, and a negative predictive value of 99.7%^17^. The high variability of the reported results may lie in the fact that the US is an operator-dependent method. In our study, we performed a correlation analysis using Pearson's correlation coefficient and proved the linear relationship between both diagnostic methods (ultrasonographic and histopathological analysis)^6^. Then, for assessing the agreement between both, the Bland-Altman analysis was performed, demonstrating a mean bias of 3.26 mm, which is considered acceptable for gallbladder size measurements^6^. An ROC curve was constructed for evaluating the diagnostic accuracy of US considering histopathological analysis as the gold standard. With an AUC obtained of 0.77 (95%CI 0.74–0.81), we can conclude that US has an acceptable discriminative ability for gallbladder polyps. Concerns may arise about the fact that ROC curves are based on comparing two diagnostic tests, and histopathological analysis is not used for diagnosis as the US^6^. Nevertheless, the definitive size of the polyps is actually determined by histopathological analysis^19^. Similar studies have been published, aiming to determine the correlation and agreement between US and histology for gallbladder polyps^16,34^. However, to the best of our knowledge, this study is the first one using all the aforementioned statical methods.

Our study determines polyp size as a predictor of the presence of adenoma. Even though the adenoma-carcinoma sequence in the gallbladder has not been well addressed yet, the available evidence suggests that at least some adenocarcinomas have arisen in pre-existing gallbladder adenomas^3,13^. There is consensus about the fact that bigger polyps are at higher risk of being neoplastic, so current guidelines recommend preemptive cholecystectomy for those patients with gallbladder polyps of 10 mm or greater^8,22,30^. In our series, the three patients diagnosed with adenocarcinoma had polyps greater than 1 cm (3.4, 2.8, and 1 cm), as measured in the preoperative US. The evidence is consistent that polyps less than 10 mm in size are very rarely associated with gallbladder carcinoma^25,26^. Nevertheless, Taskin et al. suggest that polyps smaller than 1 cm should not generate the assurance that they are non-neoplastic polyps since nearly one-third of the neoplastic polyps included in their study were actually <1cm^26^.

Polyps less than 1 cm, in general, are pseudo-polyps. Many times, they are multiple and cholesteric. The adenomas and adenocarcinomas, as exposed before, are infrequent, unique most of the time, and greater than 1 cm^26,27^. Among 127 polyps greater than 10 mm in our series, an additional imaging evaluation (magnetic resonance or computed tomography) was indicated in 18 patients, being computed tomography commonly the modality of choice. The evidence suggests that 90% of the polyps greater than 1 cm are neoplastic^8,26^. Computed tomography has been proposed for staging larger, suspicious malignant polyps and may be helpful in determining the need for cholecystectomy in this group of patients^18,24^. Satoh et al. described sensitivity, specificity, positive predictive value, negative predictive value, and diagnostic accuracy of plain CT for gallbladder carcinomas greater than 1 cm as 73, 90, 89, 75, and 81%, respectively, and demonstrated that lesion detectability on plain CT was independently associated with gallbladder carcinomas^24^. Less research has been performed looking at magnetic resonance's role in differentiating benign and malignant polyps, but some authors suggest that it may play a role in diagnosing neoplastic polyps and may be indicated for characterizing single polyps greater than 1 cm.^18,28^. However, further research is warranted to establish if computed tomography and magnetic resonance can improve the accuracy of diagnosing gallbladder polyps and avoid an unnecessary cholecystectomy. Other imaging modalities such as endoscopic US and high-resolution US have shown some promise as an adjunct to transabdominal ultrasonography, but more studies are required to assess the exact role and the category of polyps that they may provide diagnostic accuracy^18^.

Concerns arise regarding the possibility that smaller polyps may also be malignant. In this study, all adenocarcinomas were greater than 1 cm, but eight adenomas were smaller. Current guidelines recommend US follow-up for patients with polyps smaller than 1 cm^30^. The recommended follow-up interval depends on the size of the polyps and the presence of risk factors for malignancy^18,30^. However, these recommendations are based on low-quality evidence, and the correlation between the growth of the polyp and the development of malignancy has not yet been well determined^31^.

A total of 205 polyps were described as unique in the US, and among them, 84 (41%) were multiple on histopathological analysis. In contrast, 242 were described as multiple in the US, and 124 (51.2%) were unique on histopathological analysis. Finally, among the 447 patients included in the study, 52 (11.6%) did not have polyps on histopathological analysis. We hypothesized that the gallbladder's suction during cholecystectomy before extracting the operatory piece might fragment or suction the polyps and thus explain this discordance. The most common site of gallbladder retrieval in our center is through the umbilical port. When the gallbladder is found to be distended or containing large stones, it is opened at the time of the retrieval and the bile is suctioned under direct vision. This process can fragment polyps, especially cholesterol-type ones, and thus modify their number and size described in the histopathological analysis.

Our study has certain limitations. This is a retrospective study, being information bias always an issue as data were collected from institutional electronic databases. In addition, 300 patients were excluded from the analysis due to lack of data, and some US did not have the exact size of the polyps. Regarding the histopathological analysis, in approximately 15% of the cases, the biopsy showed no abnormalities. This may be due to gallbladder removal techniques during surgery, particularly aspiration of the contents as described before.

## CONCLUSION

There is a positive correlation and appropriate diagnostic accuracy between the ultrasonographic size of gallbladder polyps compared to the size of the histopathological report, with a trend to overestimate the size by about 3 mm. Neoplastic gallbladder polyps are uncommon, and it correlates with size. Gallbladder polyps of 10 mm were associated with adenomas and malignancies.
